# Analysis of Titin in Red and White Muscles: Crucial Role on Muscle Contractions Using a Fish Model

**DOI:** 10.1155/2018/5816875

**Published:** 2018-11-18

**Authors:** Ming-Ping Wu, Nen-Chung Chang, Chi-Li Chung, Wan-Chun Chiu, Cheng-Chen Hsu, Hui-Min Chen, Joen-Rong Sheu, Thanasekaran Jayakumar, Duen-Suey Chou, Tsorng-Harn Fong

**Affiliations:** ^1^Department of Pharmacology, School of Medicine, College of Medicine, Taipei Medical University, No. 250, Wuxing Street, Taipei 11031, Taiwan; ^2^Department of Obstetrics and Gynecology, Chi-Mei Medical Center, No. 901, Zhonghua Road, Yongkang District, Tainan 71004, Taiwan; ^3^Division of Cardiology, Department of Internal Medicine, Taipei Medical University Hospital, No. 252, Wuxing Street, Taipei 11031, Taiwan; ^4^Division of Pulmonary Medicine, Department of Internal Medical, Taipei Medical University Hospital, No. 252, Wuxing Street, Taipei 11031, Taiwan; ^5^School of Nutrition and Health Sciences, College of Nutrition, Taipei Medical University, No. 250, Wuxing Street, Taipei 11031, Taiwan; ^6^Department of Anatomy and Cell Biology, School of Medicine, College of Medicine, Taipei Medical University, No. 250, Wuxing Street, Taipei 11031, Taiwan

## Abstract

Several studies have compared molecular components between red and white skeletal muscles in mammals. However, mammalian skeletal muscles are composed of mixed types of muscle fibers. In the current study, we analyzed and compared the distributions of titin, lipid, phosphate ions, and fatty acid levels in red and white muscles using a fish model (*Tilapia*), which is rich in red and white muscles, and these are well separated. Oil-red O staining showed that red muscle had more-abundant lipids than did white muscle. A time-of-flight secondary-ion mass spectrometric (TOF-SIMS) analysis revealed that red muscle possessed high levels of palmitic acid and oleic acid, but white muscle contained more phosphate ions. Moreover, elastica-van Gieson (EVG) and Mito-Tracker green FM staining showed that collagen and elastic fibers were highly, respectively, distributed in connective tissues and mitochondria in red muscle. An electron micrographic analysis indicated that red muscle had a relatively higher number of mitochondria and longer sarcomere lengths and Z-line widths, while myofibril diameters were thicker in white muscle. Myofibrillar proteins separated by SDS-PAGE showed that the major giant protein, titin, was highly expressed in white muscle than in red muscle. Furthermore, ratios of titin to myosin heavy chain (MHC) (titin/MHC) were about 1.3 times higher in white muscle than red muscle. We postulated that white muscle is fit for short and strong contractile performance due to high levels of titin and condensed sarcomeres, whereas red muscle is fit for low intensity and long-lasting activity due to high levels of lipids and mitochondria and long sarcomeres.

## 1. Introduction

Skeletal muscles are highly preserved tissues in animals, are present all over the body, and form a combined network with a notable skeletal system via tendons to resist gravity and facilitate mobility. There are two major categories of muscle fibers in vertebrates: red fibers and white fibers [1]. Morphological studies showed that red fibers (slow twitch, oxidative fibers) are small in diameter and have a red color, due to their greater content of myoglobin and rich supply of capillaries. They have numerous large mitochondria beneath the sarcolemma and between the myofibrils. Lipid droplets are common in the sarcoplasm of these fibers. In contrast, white fibers (fast-twitch fibers) are larger in diameter. Their mitochondria and lipid droplets are smaller and less numerous than those of red fibers [2]. The properties of red muscle fibers make them very effective in postural maintenance, while white muscle fibers are suited for bursts of intense muscle activity [3].

In addition to morphological observations, physiologists have reported that red muscle fibers contract more slowly and are more resistant to fatigue than are white muscle fibers due to their ability to oxidatively regenerate ATP. In contrast, ATP generation in white muscle fibers depends on anaerobic glycolysis [4]. It is accepted that hydrolysis of ATP by ATPase is directly involved as the energy supply in the process of muscle contraction. Biochemical studies revealed that the ATPase activity of white muscle fibers is 3 times higher than that of red muscle fibers [5]. However, in a resting state, whether ATP is stored for use in muscle fibers and whether the stored ATP content in red muscle fibers is more or less than that in white muscle fibers are still unclear.

Titin, with a molecular mass of 3~4 MDa, is a muscle-specific elastic protein that spans from the Z-line to the M-line in the half-sarcomere; it is the largest protein known to date [6, 7]. The passive tension, elasticity, and stiffness of muscles are correlated with their titin content [8]. Previous experiments on single myofibrils prepared from the rabbit psoas muscle revealed another powerful mechanism of active force production [9]. Recent studies showed that regulation of skeletal muscle force is based on a three-filament model that includes titin, rather than a two-filament model consisting only of actin and myosin filaments [10, 11]. Titin is the third most abundant protein in muscles, after myosin and actin. Titin contents are about 10% of total myofibrillar proteins in chicken breast muscle [6] and 16% in rabbit skeletal muscle [12]. However, titin contents between red and white muscles have not been investigated in a single species.

Biochemical analyses of human muscle usually use biopsies to obtain muscle samples [13]. The optimal sample size is critical for the availability of tissue for processing. A biopsy cannot produce a large amount of muscle sample and may increase the risk of infection. On the other hand, muscles of mammals (mice, rats, rabbits, and humans) are mostly of the mixed type; that is, muscles contain both red and white muscle fibers [14, 15]. Relative proportions of the different fiber types vary among species, and in humans, they show significant variability among individuals [1]. Although we wanted to quantify and compare myofibrillar protein contents between red and white muscles, mammalian muscles which contain mixed muscle fibers cannot be precisely analyzed.

Fish are a good animal model for muscle studies, because aquatic species have a proportionally larger muscle mass. In addition, red and white muscle types in fish are distributed in different anatomical locations. The lateral triangular zone and a superficial thin layer of the trunk is a red color containing red muscle fibers, but large portions of the deep layer of the dorsal and ventral zones of the trunk are white and contain abundant white muscle fibers [16]. Moreover, white muscle fibers are associated with fast swimming behaviors in fish, such as during predation and escape, while red muscle fibers are correlated with slow movements, such as during migration and foraging [17, 18].

Zebrafish are commonly used in genetic, embryonic development, and muscle research [19]. Red and white muscles are also distributed in different region, but zebrafish are too small to separate red muscles from white muscles and obtain a sufficient amount of muscle tissues for biochemical analyses. Therefore, we used Tilapia sp., a locally common fish in Taiwan, to analyze morphological and myofibrillar proteins between red and white muscles in a single species.

The purpose of this study was to investigate morphological differences by histochemistry, time-of-flight secondary ion mass spectrometry (TOF-SIMS), and electron microscopy and quantify myofibrillar proteins, especially titin contents between red and white muscles in fish by sodium dodecyl sulfate polyacrylamide gel electrophoresis (SDS-PAGE).

## 2. Materials and Methods

### 2.1. Experimental Animals

**Figure 1:**
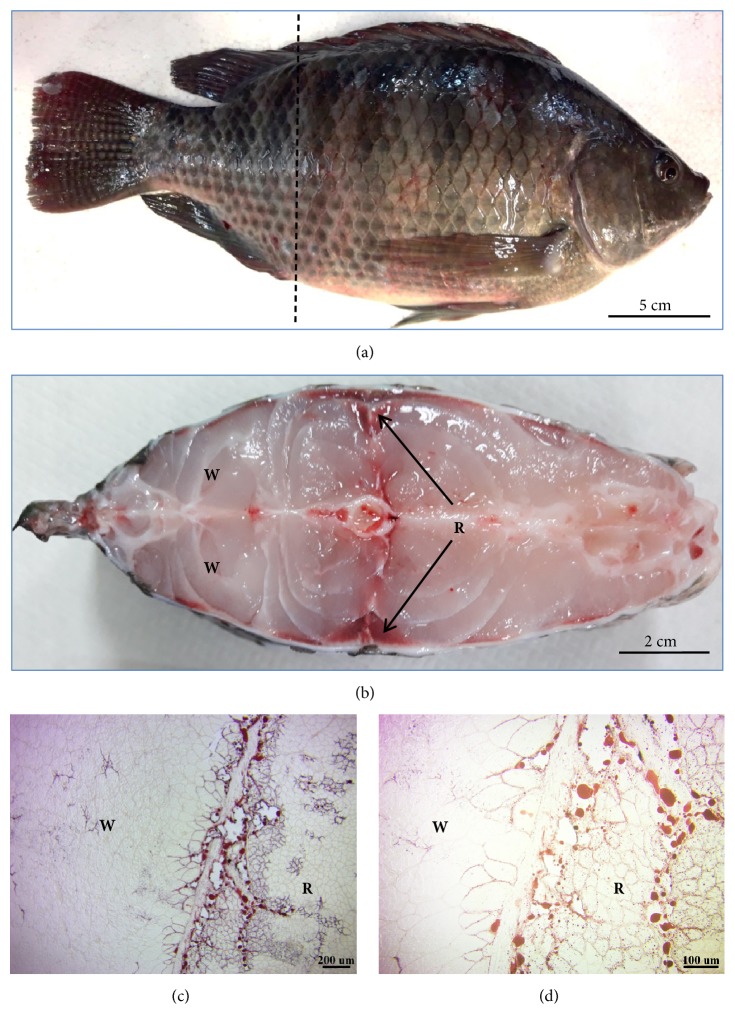
Red muscle (R) contains more lipids than white muscle (W). (a) The dotted line shows a cross-section near the cloaca of* Tilapia*. (b) Cross-section; red muscle is located on the lateral sides of the trunk (arrows), while white muscle is located on the back and ventral parts of the fish. (c) Oil-red O staining of a frozen section; red muscle contains more lipids than does white muscle. (d) More intracellular lipid droplets were seen in red muscle.

In total, 10 apparently healthy, adult male fish (*Tilapia*) (Figure 1(a)), with an average body weight of 500 ± 5 g, were purchased from a local traditional market in Taipei, Taiwan, and were transported to the laboratory live, in water. Fish were killed by cervical dislocation and then immediately dissected out. Red and white muscles were mainly located on the lateral and dorsal sides of the trunk, respectively (Figure 1(b)). The lateral part of the trunk containing a red and white muscle block (about a 1-cm^2^ area) was excised and frozen with liquid nitrogen for cryostat sectioning. All experimental protocols were approved by the Animal Ethics Committee of Taipei Medical University (license no. LAC-2017-0224).

### 2.2. Oil-Red O Staining

Oil-red O powder (Sigma-Aldrich, St. Louis, MO, USA) was dissolved in 0.5% 2-propanol (Kanto Chemical, Tokyo, Japan). The stock was then diluted to a 0.3% Oil-red O solution with distilled H_2_O and filtered through a 0.22-*μ*m filter. Frozen sections (20 *μ*m thick) of fish muscle sample were fixed with 2% paraformaldehyde and 2% glutaraldehyde (Sigma-Aldrich) in 0.1 M phosphate buffer (pH 7.4) for 10 min at room temperature. After fixation, sections were washed with phosphate buffer three times and stained with 0.3% Oil-red O solution for 10 min at room temperature. Finally, stained sections were washed with phosphate buffer three times, mounted with aqueous mounting medium, and sealed with nail polish. Sections were examined with an Olympus BH-2 light microscope (Tokyo, Japan).

### 2.3. TOF-SIMS Analysis

The TOF-SIMS analysis was carried out on a PHI TRIFT IV instrument (ULVAC-PHI, Kanagawa, Japan). This instrument was equipped with a Bi liquid metal ion gun. Cryostat sections (20 *μ*m thick) of fish muscle tissues were used for a replicate analysis in this study. The Bi_3_^+^ primary ion beam was operated at 30 keV. Surface spectra were taken from an area of 500 × 500 *μ*m in order to get an overview of the sample structure and identify ion species present at the respective surfaces. Four random areas were selected for scanning in each section, for which four spectra were separately acquired from each sample. Subsequently, negative secondary ions flying through a reflectron mass spectrometer were detected with a micro-channel plate assembly operating at 10 keV after acceleration. The high mass resolution and high mass accuracy allowed assignation of sum formulas to peaks even in the high mass range. The spectra obtained in the bunched mode were only used to identify and select peaks for imaging. Each image was normalized to the intensity of the brightest pixel. Data were collected from several image fields on each section.

### 2.4. Elastica-Van Gieson (EVG) Staining

We used the EVG staining method to examine elastic and collagen fibers in connective tissues. Frozen sections (20 *μ*m thick) of fish muscle samples were fixed with 4% paraformaldehyde in 0.1 M phosphate buffer (pH 7.4) for 10 min at room temperature. Subsequently, sections were rinsed three times with phosphate buffer for 5 min each and then sequentially stained with resorcin-fuchsin, iron hematoxylin, and van Gieson solution (purchased from Electron Microscopy Sciences, Hatfield, PA, USA) according to the manufacturer's instructions. After that, sections were dehydrated in an ethanol series. After being treated with xylene, sections were mounted with histological mounting medium. Sections were examined with an Olympus BH-2 light microscope.

### 2.5. Mito-Tracker Green FM Staining

Frozen sections (20 *μ*m thick) of fish muscle samples were incubated with 200 nM Mito-Tracker green FM (Molecular Probes, Eugene, OR, USA) for 30 min at 37°C. After washing with 0.1 M phosphate buffer (pH 7.4), sections were fixed with 4% paraformaldehyde for 10 min at room temperature. Once sections were rinsed with phosphate buffer, they were mounted with aqueous mounting medium, sealed with nail polish, and then examined with a Nikon fluorescence microscope (Tokyo, Japan).

### 2.6. Electron Microscopy (EM)

To identify ultrastructural characteristics of red and white muscles, we observed the ultrastructural morphology using EM. A piece of red or white muscle block was cut and fixed in a mixed aldehyde solution composed of 2% paraformaldehyde and 2% glutaraldehyde in 0.1 M phosphate buffer (pH 7.4). Subsequently, samples were cut longitudinally with the knife-edge parallel to the muscle fiber and postfixed using 1% osmium tetroxide in 0.1 M cacodylate buffer (pH 7.2). Samples were dehydrated in an ethanol series and embedded in Epon 812 using standard procedures. Ultrathin sections were cut and double-stained with uranyl acetate and lead citrate and then examined with a Hitachi H-600 EM (Tokyo, Japan).

### 2.7. Gel Electrophoresis and Densitometric Analysis

SDS-PAGE was performed to analyze the myofibrillar proteins actin, myosin heavy chain (MHC), and titin according to Chen et al. [20], using a 4%~12% step gradient mini-gel with an ambiguous interface. A loading range of each sample was electrophoresed on a “calibration gel.” Gel electrophoresis was performed at a constant 100 V for 2 h at room temperature until the bromophenol blue had reached approximately 5 mm above the bottom of the gel. After electrophoresis, the gel slabs were stained with Coomassie brilliant blue R-250 in 50% methanol and 10% acetic acid and subsequently destained in a 10% methanol and 10% acetic acid solution. A densitometric analysis was performed with a Photo-Print Digital Imaging System (IP-008-SD; Vilber Lourmat, Cedex, France) with Bio-1D analytic software. The optical density integrals (ODIs) of MHC and titin were measured for each loading volume, and the slope of the relationship between the ODI and loading volume was determined by a linear regression analysis. Also, the ratio of titin to MHC (titin/MHC) was calculated as the slope of the titin ODI/loading volume divided by the slope of the MHC ODI/loading volume [21]. Prestained Protein Ladder # PREP1025 (Bioman Scientific, Taipei, Taiwan) was used as a molecular weight reference.

### 2.8. Statistical Analysis

Values are expressed as the mean ± standard error of the mean (SEM). Student's paired* t*-test was used to verify the significance of the differences between red and white muscles. At all times,* p*<0.05 was considered significant.

## 3. Results

### 3.1. Characteristics of Red and White Muscles of Tilapia

In a cross-section of tilapia, the red and white muscles were easily identified according to the muscle color and location. As shown in Figure 1(b), white muscle was abundant in the dorsal and ventral parts of the trunk, while red muscle was triangular and located on the lateral side of the trunk. Oil-red O staining showed that red muscle contained more lipid droplets than did white muscle (Figures 1(c) and 1(d)).

### 3.2. TOF-SIMS Analysis of Red and White Muscles

**Figure 2:**
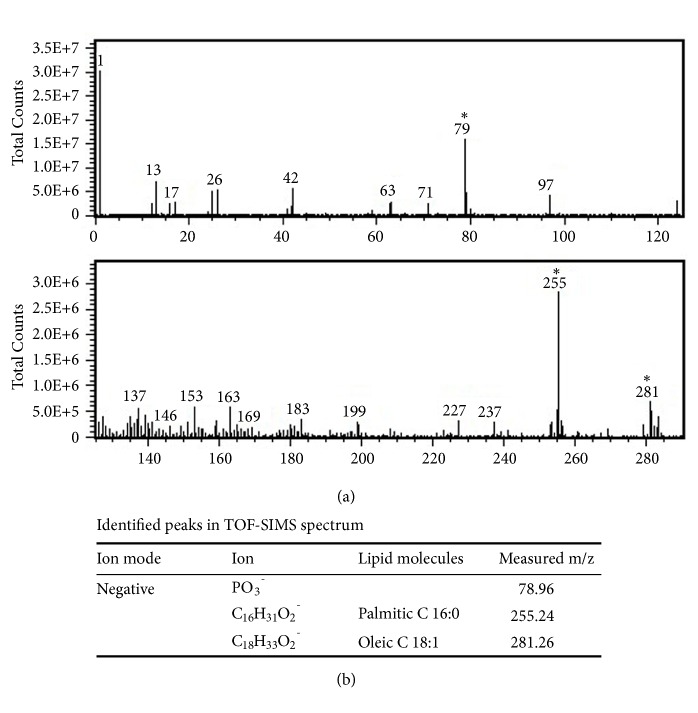
Negative ion TOF-SIMS mass spectrum. (a) Partial mass spectrum obtained from a region of interest containing red and white muscles in a fish muscle section in the m/z range of 0~300. (b) Values of* m/z* of identified peak areas are labeled in the TOF-SIMS spectrum.

**Figure 3:**
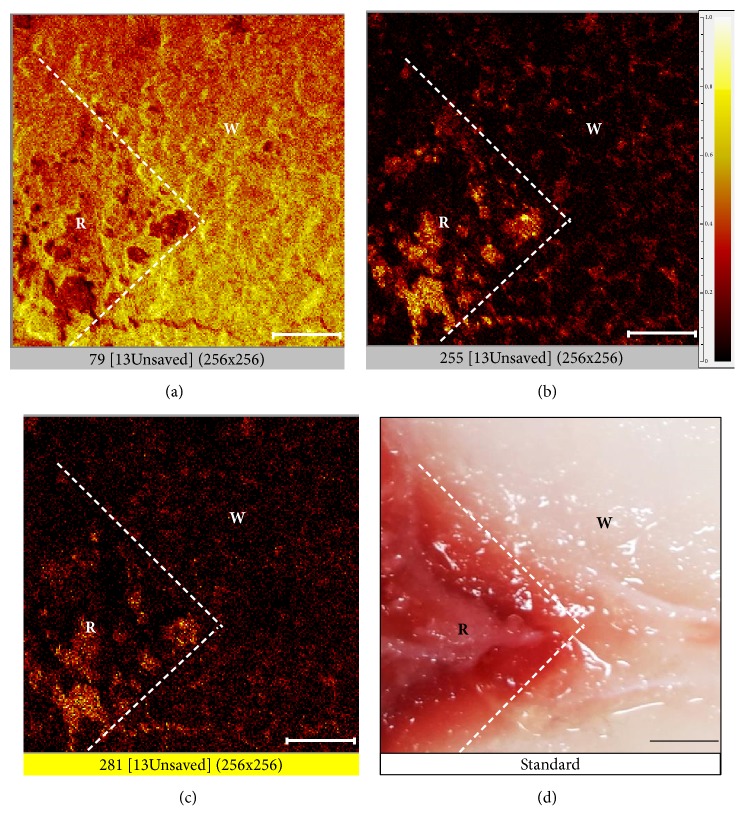
TOF-SIMS images of negative ion and fatty acid compositions of red (R) and white (W) muscles. (a) TOF-SIMS ion image showing the distribution of phosphate (PO^3−^) m/z 79. (b) TOF-SIMS ion image showing the distribution of palmitic acid m/z 255. (c) TOF-SIMS ion image showing the distribution of oleic acid m/z 281. (d) Bright-field standard. Bar: 100 *μ*m. Image size 500 × 500 *μ*m (256 × 256 pixels).

Results of the TOF-SIMS spectral analysis are shown in Figure 2(a), and the respective identified peaks of phosphate ions (PO_3_^−^,* m/z* 79.96), palmitic acid (18:0,* m/z* 255.24), and oleic acid (18:1,* m/z* 281.26) are shown in Figure 2(b). TOF-SIMS also imaged the distribution of inorganic ions and fatty acids in red and white muscles of tilapia. This analysis produced an interesting image in which phosphate ions (PO_3_^−^,* m/z* 79) were found to be more enriched in white muscle (Figure 3(a)) than in red muscle. In contrast, palmitic acid (18:0,* m/z* 255) and oleic acid (18:1,* m/z* 281) showed higher levels in red muscle compared to white muscle (Figures 3(b) and 3(c)). Moreover, the distribution pattern of palmitic acid was similar to that of oleic acid in red muscle.  Figure 3(d) shows areas of red and white muscles used as standards for the TOF-SIMS analysis.

### 3.3. Elastic and Collagen Fibers in Connective Tissues of Red and White Muscles

**Figure 4:**
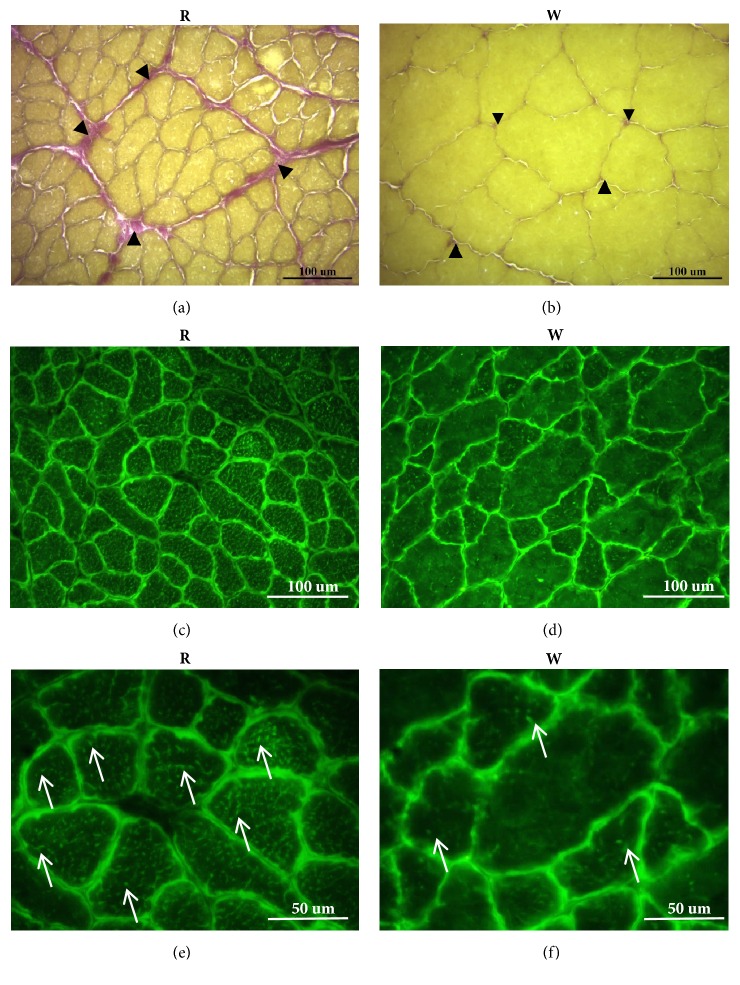
Elastica-van Gieson (EVG) and Mito-Tracker Green FM staining to, respectively, detect connective tissues and mitochondria. (a) and (b) EVG staining labeled connective tissue between muscle fibers. Collagen fibers are red, and elastic fibers are purple. There was more connective tissue (arrowheads) between muscle fibers in red muscle (R) than white muscle (W). In addition, the size of red muscle fibers was smaller than that of white muscle fibers. (c–f) Mito-Tracker Green FM staining to label intracellular mitochondria. (c) and (e) show the mitochondrial distribution images of red muscle. (d) and (f) show the mitochondrial distribution images of white muscle. There was a high density of green fluorescence surrounding each muscle fiber in both muscles, indicating that abundant mitochondria were located beneath the sarcolemma. In addition, more green dots or trabecula (arrows) were found in red muscle fibers than in white muscle fibers ((e) and (f)), indicating that more-abundant mitochondria were located between myofibrils in red muscle than in white muscle.

We used the EVG staining method to examine elastic fibers and collagen fibers in connective tissues. Sections showed a high level of connective tissues between red muscle fibers (Figure 4(a)). The size of white muscle fibers was obviously larger than red muscle fibers (Figure 4(b)). The EVG staining image clearly indicated that red muscle contained more connective tissue fibers, such as collagen and elastic fibers, than did white muscle (Figures 4(a) and 4(b)).

### 3.4. Mitochondrion Localization of Red and White Muscle Fibers

Localization of mitochondria in red and white muscles was determined using Mito-Tracker green FM staining. Intracellular mitochondria were stained green, whereas connective tissues between muscle fibers were black (Figures 4(c)–4(f)). Results showed that abundant mitochondria accumulated beneath the sarcolemma of both red and white muscle fibers. However, more mitochondria between myofibrils were found in red muscle fibers than in white muscle fibers (arrows in Figures 4(e) and 4(f)).

### 3.5. EM Ultrastructure of Red and White Muscle Fibers

**Figure 5:**
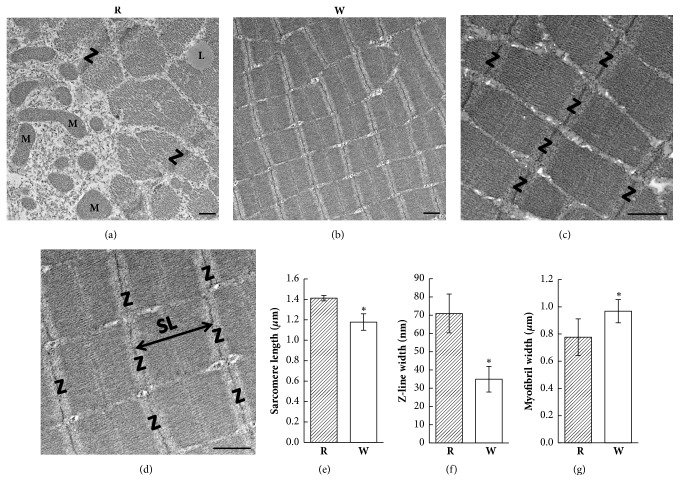
Electron microscopic (EM) ultrastructural images of red (R) and white (W) muscle fibers. (a) Several mitochondria are located near the sarcolemma. Lipid droplets can be seen between the myofibrils in red muscle fibers. (b) EM image indicating less cytoplasm and fewer mitochondria between myofibrils in white muscle fibers. (c) A longitudinal section displayed the well-organized sarcomere in red muscle fibers. (d) Well-organized sarcomeres were also observed in white muscle fibers. The sarcomere length (double-headed arrow) is the distance between two Z-lines. (e) The sarcomere length was shorter in white muscle fibers. (f) The Z-line width was thinner in white muscle fibers. (g) The myofibril width was larger in white muscle fibers. *∗ p* < 0.001. L, lipid droplet; M, mitochondria; SL, sarcomere length; Z, Z-line. Bar: 0.5 *μ*m.

Red and white muscle fibers were distinguishable in longitudinal EM sections (Figure 5). Red muscle fibers contained numerous large mitochondria and lipid droplets beneath the sarcolemma and between myofibrils (Figure 5(a)); however, white fibers rarely had mitochondria or lipid droplets (Figure 5(b)). The sarcomere length between two Z-lines was longer in red muscle fibers (1.411 ± 0.026 *μ*m) than white muscle fibers (1.178 ± 0.081 *μ*m) (Figures 5(c)–5(e)). The width of the Z-line of red muscle fibers (70.9 ± 10.6 nm) was nearly double that of white muscle fibers (35.0 ± 7.0 nm) (Figures 5(c), 5(d), and 5(f)). The average diameter of white muscle myofibrils was relatively larger (0.968 ± 0.085 *μ*m) than that of red muscle myofibrils (0.776 ± 0.135 *μ*m) (Figures 5(c), 5(d), and 5(g)).

### 3.6. Myofibrillar Proteins in Red and White Muscles

**Figure 6:**
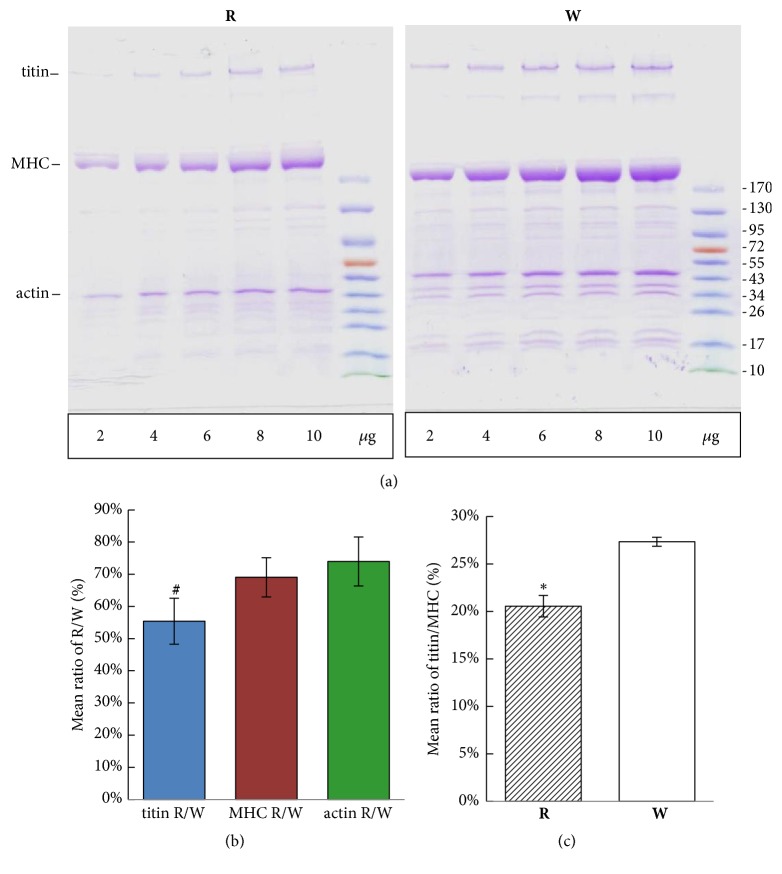
SDS-PAGE protein analysis of red (R) and white (W) muscles. (a) After loading equal concentrations (2~10 *μ*g) of total proteins, white muscle displayed more myofibrillar proteins than did red muscle. Titin, myosin heavy chain (MHC), and actin were the three major myofibrillar proteins. (b) Ratios of titin, MHC, and actin in red muscle to white muscle. (c) Mean ratios of titin/MHC in red and white muscles. ^#^* p* < 0.01, *∗ p *< 0.001.

SDS-PAGE was performed to analyze myofibrillar proteins in red and white muscles using 4%~12% step gradient mini-gels. Titin, MHC, and actin were all visible in Coomassie brilliant blue-stained step gradient mini-gels (Figure 6(a)). Relative proportions of myofibrillar proteins were calculated by quantitative densitometry. A linear regression analysis between the ODI and loading volume showed that slopes of the ODI/loading volume of titin were 2144.1 ± 47.5 and 4090.3 ± 397.8 in red and white muscles, respectively. Thus, the ratio of titin in red to white muscle (titin R/W) was about 54.6% ± 7.4% which was significantly lower than those of MHC (MHC R/W was 69.3% ± 5.0%) and actin (actin R/W was 74.0% ± 7.6%) (Figure 6(b)). Ratios of titin to MHC (titin/MHC) were 20.6% ± 1.1% and 27.3% ± 0.5% in red and white muscles, respectively (Figure 6(c)). These results show that the titin protein was more abundant in white muscle than red muscle.

## 4. Discussion

In this study, we used the fish (*Tilapia*) to analyze the ATP, titin, and sarcomere ultrastructure by using novel TOF-SIMS and EM techniques in red and white muscles. Results of Oil-red O staining showed that red muscle contained more lipid droplets than did white muscle. The spectra and images of TOF-SIMS demonstrated that phosphate ions were rich in white muscles, but more fatty acids (palmitic acid and oleic acid) were found in red muscles. In addition, collagen and elastic fibers of connective tissue were more enriched in red muscle than in white muscle. Despite both muscles containing mitochondria beneath the sarcolemma, numerous long and large mitochondria between the myofibrils were only seen in red muscle. Moreover, ultrastructural observations determined that the sarcomere length and Z-line width were larger in red muscle, but the myofibril diameter was thicker in white muscle. Finally, white muscle had more myofibrillar proteins (titin, MHC, and actin) than did red muscle. The ratio of titin to myosin (titin/MHC) was lower in red muscle than in white muscle. According to these findings, we proposed that white muscle has faster and more-powerful contractions than red muscle, which may result from high levels of titin and phosphate ions, a source of ATP molecules, but less connective tissue and a shorter sarcomere length.

Previous biochemical, histochemical, and EM studies showed up to a threefold higher lipid content in red muscle fibers than in white muscle fibers obtained from a muscle biopsy sample of healthy subjects [22]. Those data were supported by data using Oil-red O and immunofluorescence microscopy [23]. Characteristics of red muscles in fish are also a good capillary supply and high levels of mitochondria, lipid droplets, and glycogen stores [24]. Consistent with those studies, the present work also found that red muscle contains higher numbers of mitochondria and lipid droplets. In addition, we found that red muscle contains high amounts of palmitic acid (18:0,* m/z* 255) and oleic acid (18:1,* m/z* 281) compared to white muscle. TOF-SIMS images showed that the distribution pattern of palmitic acid was similar to that of oleic acid, suggesting that lipid droplets in red muscle contain both fatty acids. These fatty acids in red muscle might be the fuel for the oxidative regeneration of ATP, like glycogen in white muscle is the source of anaerobic glycolysis for ATP generation [25]. A previous EM study by Nag [5] showed that lipid droplets in red muscle fibers were closely associated with mitochondria. Red muscle fibers are mainly used for posture maintenance and sustained energy-efficient exercise. These studies suggest that red muscle fibers are resistant to fatigue because of their ability to oxidatively regenerate ATP.

The level of phosphate ions, an actual energy source of ATP, was higher in white muscle than red muscle in a resting condition. Available source of ATP at rest can quickly provide energy for contractile activity in white muscles for burst movement [26, 27]. After depleting stored ATP, its regeneration is supported by creatine kinase, adenylate kinase, and AMP-activated protein kinase (AMPK) [28]. Muscle contractions during exercise were found to increase AMPK activity and enhance the immediate availability of both carbohydrates and fats as fuel for mitochondrial oxidation and increased rates of ATP production [29, 30]. Another source of ATP generation is the glycolytic pathway which is more effective in white muscle than in red muscle [31]. Furthermore, it is well known that ATP is regenerated during the tricarboxylic acid cycle in mitochondria, and this process is more effective in red muscle than in white muscle [32]. In contrast, white muscle contained few mitochondria and lipid droplets, suggesting that white muscle fatigues rapidly. Ultrastructural observations demonstrated that white muscle had shorter sarcomere lengths and wider myofibril diameters than those of red muscle, indicating that white muscle could exhibit stronger and faster contractions than red muscle.

On the other hand, the generally accepted mechanism of active force production in a sarcomere is based on the actin and myosin filament sliding model, the so-called cross-bridge theory [33]. Briefly, myosin heads attach to actin filaments and pull the actin filaments towards the M-line in the center of the sarcomere. Thereby the sarcomere shortens and produces an active force. The discovery of the giant elastic protein, titin, dramatically expanded our understanding of muscle structure and function [6, 8, 34]. In addition, titin also plays an important role as a molecular scaffold for thick and presumably thin filament formation during myofibrillogenesis [35]. It is because of this supportive role that titin damage results in abnormal sarcomeric organization and myofibril disassembly [36, 37]. Considering these important functions of titin, we characterized muscle proteins in tilapia and found that titin was more highly expressed in white muscle than red muscle. Titin contents were about 10% of total myofibrillar proteins in chicken breast muscle [6], 16% in rabbit skeletal muscle, and 13% in fish muscle [12]. Ratios of titin relative to myosin were estimated to be 1:9.5 for rabbit cardiac muscle and 1:4.2 for rabbit skeletal muscle [38]. In this study, ratios of titin to myosin were estimated to be 0.206 (1:4.8) for red muscle and 0.273 (1:3.7) for white muscle. White muscle containing a higher titin/MHC ratio represents a more-elastic protein in sarcomere units. Since titin is the main passive tension source of muscles, white muscle is relatively more rigid than red muscle. In addition, skeletal muscle force regulation highly depends on titin filaments, rather than actin and myosin filaments [10, 11]. White muscle contracts faster and more efficiently than red muscle, which might result from a higher level of titin and more-organized sarcomeres.

Studies have demonstrated that titin stiffness increases in activated muscle prior to development of force [39] and also demonstrated a role for titin in residual force enhancement [40]. Another study suggested that titin binds calcium upon activation, thereby increasing its spring stiffness, and that some proximal part of titin may bind to actin, thereby potentially decreasing titin's free spring length in the I-band region, thus possibly increasing titin's stiffness and its force [9]. Given the weight of these evidences, it is now more ungenerous to presume that not only cross-bridges, but also titin contributes to dynamic force production.

## 5. Conclusion

The present study was carried out to discover the biochemical characteristics which are important in muscle contraction in red and white muscles of fish (*Tilapia*). Our results suggest that white muscle contains higher levels of titin and phosphate ions and shorter sarcomere lengths, which may be involved in faster and more-powerful muscle contractions. In addition, red muscle was enriched in well-balanced lipid droplets, fatty acids, and mitochondria and had longer sarcomere lengths for slow, long-term contractions. Taken together, the present results of titin and energetic parameters in red and white muscles provide evidence that white muscle is more suitable for short and powerful contractile performance than red muscle.

## Data Availability

The data used to support the findings of this study are included within the article.

## References

[1] Schiaffino S., Reggiani C. (2011). Fiber types in Mammalian skeletal muscles. *Physiological Reviews*.

[2] Ogata T. (1988). Structure of Motor Endplates in the Different Fiber Types of Vertebrate Skeletal Muscles. *Archives of Histology and Cytology*.

[3] Franzini-Armstrong C., Peachey L. D. (1981). Striated muscle-contractile and control mechanisms.. *The Journal of Cell Biology*.

[4] Huang Y.-C., Dennis R. G., Baar K. (2006). Cultured slow vs. fast skeletal muscle cells differ in physiology and responsiveness to stimulation. *American Journal of Physiology-Cell Physiology*.

[5] Nag A. C. (1972). Ultrastructure and adenosine triphosphatase activity of red and white muscle fibers of the caudal region of a fish, salmo gairdneri. *The Journal of Cell Biology*.

[6] Wang K., McClure J., Tu A. (1979). Titin: major myofibrillar components of striated muscle. *Proceedings of the National Acadamy of Sciences of the United States of America*.

[7] Granzier H., Labeit S. (2007). Structure-function relations of the giant elastic protein titin in striated and smooth muscle cells. *Muscle & Nerve*.

[8] Lindstedt S., Nishikawa K. (2017). Huxleys' Missing Filament: Form and Function of Titin in Vertebrate Striated Muscle. *Annual Review of Physiology*.

[9] Leonard T. R., Herzog W. (2010). Regulation of muscle force in the absence of actin-myosin-based cross-bridge interaction. *American Journal of Physiology-Cell Physiology*.

[10] Herzog W., Leonard T., Joumaa V., DuVall M., Panchangam A. (2012). The three filament model of skeletal muscle stability and force production. *Molecular & Cellular Biomechanics*.

[11] Schappacher-Tilp G., Leonard T., Desch G., Herzog W. (2015). A novel three-filament model of force generation in eccentric contraction of skeletal muscles. *PLoS ONE*.

[12] Seki N., Watanabe T. (1984). Connectin content and its post-mortem changes in fish muscle. *The Journal of Biochemistry*.

[13] Melendez M. M., Vosswinkel J. A., Shapiro M. J. (2007). Wall Suction Applied to Needle Muscle Biopsy-A Novel Technique for Increasing Sample Size. *Journal of Surgical Research*.

[14] Ali Khan M. (1976). Histochemical characteristics of vertebrate striated muscle: A review. *Progress in Histochemistry and Cytochemistry*.

[15] Tunell G. L., Hart M. N. (1977). Simultaneous Determination of Skeletal Muscle Fiber, Types I, IIA, and IIB by Histochemistry. *JAMA Neurology*.

[16] Wakeling J. M., Johnston J. A. (1999). White strain the carp red to white muscle gearing ratios in fish. *The Journal of Experimental Biology*.

[17] SäNger A., Stoiber W. (2001). Muscle Fiber Diversity and Plasticity. *Muscle Development and Growth*.

[18] Martin-Perez M., Fernandez-Borras J., Ibarz A. (2012). New insights into fish swimming: A proteomic and isotopic approach in gilthead sea bream. *Journal of Proteome Research*.

[19] Hasumura T., Meguro S. (2016). Exercise quantity-dependent muscle hypertrophy in adult zebrafish (Danio rerio). *Journal of Comparative Physiology B: Biochemical, Systemic, and Environmental Physiology*.

[20] Chen S.-P., Sheu J.-R., Lai C.-Y., Lin T.-Y., Hsiao G., Fong T.-H. (2005). Detection of myofibrillar proteins using a step gradient minigel with an ambiguous interface. *Analytical Biochemistry*.

[21] Wei J.-H., Chang N.-C., Chen S.-P., Geraldine P., Jayakumar T., Fong T.-H. (2015). Comparative decline of the protein profiles of nebulin in response to denervation in skeletal muscle. *Biochemical and Biophysical Research Communications*.

[22] Essén B., Jansson E., Henriksson J., Taylor A. W., Saltin B. (1975). Metabolic Characteristics of Fibre Types in Human Skeletal Muscle. *Acta Physiologica Scandinavica*.

[23] van Loon L. J. C., Koopman R., Stegen J. H. C. H., Wagenmakers A. J. M., Keizer H. A., Saris W. H. M. (2003). Intramyocellular lipids form an important substrate source during moderate intensity exercise in endurance-trained males in a fasted state. *The Journal of Physiology*.

[24] Kiessling A., Ruohonen K., Bjørnevik M. (2006). Muscle fibre growth and quality in fish. *Archiv Tierzucht/Archives Animal Breeding*.

[25] Kiens B. (2006). Skeletal muscle lipid metabolism in exercise and insulin resistance. *Physiological Reviews*.

[26] Barclay C. J., Constable J. K., Gibbs C. L. (1993). Energetics of fast‐ and slow‐twitch muscles of the mouse.. *The Journal of Physiology*.

[27] Blei M. L., Conley K. E., Kushmerick M. J. (1993). Separate measures of ATP utilization and recovery in human skeletal muscle.. *The Journal of Physiology*.

[28] Winder W. W., Thomson D. M. (2007). Cellular energy sensing and signaling by AMP-activated protein kinase. *Cell Biochemistry and Biophysics*.

[29] Vavvas D., Apazidis A., Saha A. K. (1997). Contraction-induced changes in acetyl-CoA carboxylase and 5′-AMP- activated kinase in skeletal muscle. *The Journal of Biological Chemistry*.

[30] Wojtaszewski J. F. P., MacDonald C., Nielsen J. N. (2003). Regulation of 5′-AMP-activated protein kinase activity and substrate utilization in exercising human skeletal muscle. *American Journal of Physiology-Endocrinology and Metabolism*.

[31] Spamer C., Pette D. (1977). Activity patterns of phosphofructokinase, glyceraldehydephosphate dehydrogenase, lactate dehydrogenase and malate dehydrogenase in microdissected fast and slow fibres from rabbit psoas and soleus muscle. *Journal of Histochemistry & Cytochemistry*.

[32] Leary S. C., Lyons C. N., Rosenberger A. G., Ballantyne J. S., Stillman J., Moyes C. D. (2003). Fiber-type differences in muscle mitochondrial profiles. *American Journal of Physiology-Regulatory, Integrative and Comparative Physiology*.

[33] Huxley A. F., Niedergerke R. (1954). Structural changes in muscle during contraction: interference microscopy of living muscle fibres. *Nature*.

[34] Maruyama K. (1976). Connectin, an elastic protein from myofibrils. *The Journal of Biochemistry*.

[35] Gregorio C. C., Granzier H., Sorimachi H., Labeit S. (1999). Muscle assembly: a titanic achievement?. *Current Opinion in Cell Biology*.

[36] Van Der Ven P. F. M., Bartsch J. W., Gautel M., Jockusch H., Fürst D. O. (2000). A functional knock-out of titin results in defective myofibril assembly. *Journal of Cell Science*.

[37] Udaka J., Ohmori S., Terui T. (2008). Disuse-induced preferential loss of the giant protein titin depresses muscle performance via abnormal sarcomeric organization. *The Journal of General Physiology*.

[38] Suzuki J., Kimura S., Maruyama K. (1993). Connectin content in rabbit cardiac and skeletal muscle. *International Journal of Biochemistry*.

[39] Cornachione A. S., Leite F., Bagni M. A., Rassier D. E. (2016). The increase in non-cross-bridge forces after stretch of activated striated muscle is related to titin isoforms. *American Journal of Physiology-Cell Physiology*.

[40] Herzog W., Schappacher G., DuVall M., Leonard T. R., Herzog J. A. (2016). Residual force enhancement following eccentric contractions: A new mechanism involving titin. *Physiology Journal*.

